# Roadsides provide refuge for orchids: characteristic of the surrounding landscape

**DOI:** 10.1002/ece3.6920

**Published:** 2020-10-26

**Authors:** Réka Fekete, Judit Bódis, Bence Fülöp, Kristóf Süveges, Renáta Urgyán, Tamás Malkócs, Orsolya Vincze, Luís Silva, Attila Molnár V.

**Affiliations:** ^1^ Department of Botany University of Debrecen Debrecen Hungary; ^2^ Department of Plant Sciences and Biotechnology, Georgikon Campus Szent István University Keszthely Hungary; ^3^ Balaton‐felvidéki National Park Directorate Csopak Hungary; ^4^ Wetland Ecology Research Group Department of Tisza Research Centre for Ecological Research‐DRI Debrecen Hungary; ^5^ Evolutionary Ecology Group Hungarian Department of Biology and Ecology Babeş‐Bolyai University Cluj Napoca Romania; ^6^ Faculty of Sciences and Technology University of Azores Ponta Delgada Portugal; ^7^ InBIO Research Network in Biodiversity and Evolutionary Biology CIBIO‐Açores University of the Azores Ponta Delgada Portugal

**Keywords:** anthropogenic habitats, ecological corridor, landscape matrix, linear landscape elements, Orchidaceae, roadside

## Abstract

Seminatural habitats are declining throughout the world; thus, the role of small anthropogenic habitats in the preservation of plants is becoming increasingly appreciated. Here, we surveyed the orchid flora of roadside verges in five Central European countries (Austria, Hungary, Romania, Slovakia, and Slovenia) and tested how the surrounding landscape matrix affects the overall number of species and individuals, and also different functional groups of orchids. We found more than 2,000 individuals of 27 orchid species during our surveys. According to our results, the increasing coverage of agricultural and urban areas negatively affects both the number of orchid species and individuals on roadsides. Our study further suggests that differences in the surrounding habitats affect which species are found on roadsides, since the increasing coverage of grasslands or forested areas around orchid occurrences had a significant positive effect on the number of grassland or forest‐dwelling species and individuals, respectively. Most variance in orchid numerosity and diversity was explained by the cover of the suitable habitat types of the respective taxa in the surrounding landscape of the sampling points. This highlights the importance of roadsides acting as refugia for numerous species and valuable plant communities as well as in supporting biodiversity in general.

## Graphical abstract

Increasing agricultural and urban areas negatively affects orchids on remaining habitat islands, such as roadside verges. But the fact that numerous orchid species and individuals are inhabiting these linear meadows suggests that these habitats are important for their conservation.

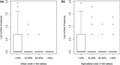

## 1 INTRODUCTION

Seminatural habitats are dominated by native flora, characterized by a typical diversity and species composition, but they bear human‐induced alterations. Despite disturbance, they generally host a large number of threatened plant species and have long been considered as hotspots for biodiversity (Benton et al., 2003; Henle et al., 2008). During the last century, intensification of human activities has led to a dramatic reduction of seminatural habitats, contributing to the severe decline of biodiversity worldwide (Butchart et al., 2010; Hooftman & Bullock, 2012; Malcolm & Markham, 2000). Seminatural habitats are subject to several threats, such as habitat degradation, destruction and fragmentation (Nascimbene et al., 2016; Tikka et al., 2000; Tilman et al., 2001), intensification of agricultural land use, and abandonment of traditional agricultural practices (Bignal & McCracken, 1996). For instance, seminatural grasslands are often converted into arable lands in response to a higher demand for food production (Hodgson et al., 2005) and afforested for timber production (Mason, 2007), or they are frequently lost to urbanization (Feranec et al., 2010). These alterations to natural and seminatural habitats urge the shift of focus of conservation‐oriented research toward anthropogenic habitats that have a potential to provide refuge for native flora elements. Several anthropogenic habitats were shown to provide such refuges, including cemeteries, poplar plantations, orchards, and roadsides (Bódis et al., 2018; Djordjević & Tsiftsis, 2020; Fekete et al., 2017; Löki et al., 2015; Süveges et al., 2019).

Road constructions are among the most widespread modifications to natural landscapes, which have intensified in both frequency and structural complexity during the last century (Ascensão et al., 2018; Bennett, 1991; Noss & Cooperrider, 1994). Roads impose numerous negative effects on natural ecosystems, including light and sound pollution, introduction of novel mortality risk factors (e.g., collision with vehicles), imposing barriers to dispersal, inducing alterations to the behavior of animals, and their physical and chemical environment, but they also contribute to the spread of exotic species (Trombulak & Frissell, 2000). Despite these negative effects, the beneficial role of roadsides as linear landscape elements is increasingly appreciated. For instance, roadsides appear to serve as refuges in the landscape and aid the maintenance of plant species richness, and have been considered as important areas due to their function in supporting native vegetation in Brazil (Allem, 1997), Pakistan (Ahmad et al., 2009; Akbar et al., 2003), Saudi Arabia (Batanouny, 1979), Australia (Bennett, 1991; Hussey, 1999; Schabel & Eldridge, 2001), South Africa (Dawson, 1991), and several regions across Europe, including Belgium (Deckers et al., 2005; Godefroid, 1999), Finland (Jantunen et al., 2006), Norway (Hovd & Skogen, 2005), Sweden (Cousins, 2006), the Balkans, the Eastern Mediterranean region (Djordjević & Tsiftsis, 2020; Fekete et al., 2017, 2019), and the United Kingdom (Atherden, Rotherham, & Handley, 2018; Harrington, 1994; Perring, 1969; Way, 1970).

Given that appropriate management practices are adopted, road verges might allow the persistence of valuable grassland communities, as well as the maintenance of rare species, conveying a significant conservation value to these man‐made habitats (Auestad et al., 2011; Hovd & Skogen, 2005). In some regions, roadside habitats have even been regarded as “Roadside Nature Reserves” and they receive special management due to their recognized conservation priority (Dawson, 1991; Parr & Way, 1988; Spooner, 2005). In the United Kingdom, for instance, almost half of the native plant species can be found on roadsides (Way, 1970). Moreover, even regularly mowed roadsides can serve as refuges for endangered grassland species, for instance, the highly cut‐tolerant *Gentianella campestris* in Finland (Huhta & Rautio, 2007).

Roadsides not only provide refuge to native flora, but were shown to serve as ecological corridors for a wide range of taxa (Gustafsson & Hansson, 1997; Haddad et al., 2003). Nonetheless, at least in the case of plants, the ecological corridor role of roads is most widely demonstrated by the dispersal of alien and invasive species alongside them (Bacaro et al., 2015; Das & Duarah, 2013; Gulezian et al., 2012; Joly et al., 2011; Lin, 2007). Ecological or landscape corridors are strips of suitable habitats connecting isolated habitat patches and are considered to facilitate gene flow and the movement of species between these, thus increasing the number of native species in large‐scale communities and reducing the negative effects of fragmentation (Cody et al., 1975; Damschen et al., 2006). Creating ecological corridors recently became a widespread ecological management practice; nonetheless, some studies question their effectiveness in aiding the movement of organisms between otherwise fragmented habitats (Beier & Noss, 1998; Hobbs, 1992; Merriam & Saunders, 1993; Simberloff et al., 1992). A survey by Tikka et al. (2001) suggests that roadsides play a direct role in the dispersal of grassland communities, clearly serving as ecological corridors. Nonetheless, Fritz and Merriam (1993) found no support of the role of fencerows serving as ecological corridors of forest floor herbs. A number of recent studies suggest a rather indirect impact of linear landscape elements on the spread of taxa, by facilitating pollination and seed dispersal between suitable habitats or by promoting plant–animal interactions (Haddad et al., 2003; Tewksbury et al., 2002; Van Rossum & Triest, 2012). According to previous studies, there could be a difference between grassland and woodland plant species in the use of ecological corridors. For instance, grassland species can easily spread to alternative open habitats, such as roadsides due to regular mowing of verges which keeps the vegetation low, but in the case of woodland species, the use of corridors is more difficult (Fritz & Merriam, 1993; Tikka et al., 2001). In the case of roadsides, the dispersal of small seeds can also be facilitated by the air turbulence of cars (Ross, 1986) or by the mud attached to the vehicles, which often contain large number of seeds, especially when the roadside vegetation is well developed (Clifford, 1959). Recent studies highlight the role of vehicles in the spread of alien species (Von Khan et al., 2018han et al., 2018; Nguyen, 2011; der Lippe & Kowarik, 2007).

Although roadsides are often isolated remnants of seminatural habitats, their species richness is largely dependent on the surrounding landscape (Cousins, 2006). For instance, Cousins and Lindborg (2008) noted that in the most intensively managed landscapes, the species richness of roadsides and midfield islets declines with increasing distance to seminatural grasslands.

In order to investigate the role of roads as refugia in the context of the surrounding landscape, we performed a systematic study of the flora of roadsides, focusing in particular on orchids as model organisms. Orchids rely greatly on pollinators and mycorrhizal fungi (Waterman & Bidartondo, 2008); thus, they are good indicators of overall local biodiversity (Swarts & Dixon, 2009). Moreover, colonization by orchids has long been known in roadsides (Turrill, 1932) as well as in other anthropogenically strongly influenced habitats (Box , 1999; Bzdon, 2009; Deák et al., 2016; Esfeld et al., 2008; Grant & Koch, 2003; Jurkiewicz et al., 2001; Kelcey, 1984; Löki et al., 2015; Lundholm & Richardson, 2010; Molnár V. et al., 2017; Ratcliffe, 1974). For instance, in the Mediterranean region, orchids are frequently present on roadsides (Brandes, 1998a, 1998b; Fekete et al., 2019), *Himantoglossum* (lizard orchids) being one of the most characteristic genera utilizing these anthropogenic habitats (Bódis et al., 2018; Federici & Serpieri, 1868; Fekete et al., 2017; Good, 1936; Klaver, 2011; Zahariev, 2014).

The central aims of the current study were to (a) assess species and individual numbers using roadsides as habitats in Central European countries and (b) examine how the landscape matrix affects the species composition, diversity, and abundance of orchids generally and also different functional groups of orchids in the sampled habitats.

## 2 MATERIALS AND METHODS

### 2.1 Fieldwork

Field sampling was carried out in five Central European countries (Austria, Hungary, Romania, Slovakia, and Slovenia). Two types of sampling processes were adopted. First, we conducted thematic sampling by driving along asphalt roads and we stopped in every 5 km. Second, we conducted non‐thematic sampling, meaning that we stopped at every road section, where orchids were spotted from the car while driving. Details of the sampling localities and sampling periods are given in Table 1. At every sampling point, we recorded geo‐coordinates (WGS84 format) and altitude (m) using a Garmin eTrex Legend GPS Device. Where orchids were present, we additionally recorded the list of orchid species and the number of specimens belonging to each of these along a 50‐m road section on one side of the road. The width of the surveyed area usually spanned from 0 to 10 m, being delimited by roadway on one side and ditches, walls, or taller vegetation on the other side. In some cases, identification of orchids to the species level was not possible, due to their vegetative phenological state. In the latter case, we counted the number of individuals, but these were only included in the overall count of orchid individuals. Taxa were identified following Delforge (2006), and the nomenclature used in this work follows this source.

**TABLE 1:** Number of the different sampling points and the date of the surveys carried out in the sampled countries

Country	Number of non‐thematic sampling points	Number of thematic sampling points	Survey period
Austria	2	50	14–15 July 2018
Hungary	27	156	8 July 2015
			3–6 May 2017
			2–3 May 2018
			11–13 May 2018
Romania	4	92	17–20 June 2017
Slovakia	1	91	27–30 May 2017
Slovenia	1	76	11–13 July 2018

### 2.2 Landscape variables

For each sampling point, we calculated land cover variables, based on the surrounding landscape matrix. For this, we used the 2018 CORINE Land Cover (CLC) dataset (available via the Copernicus Land Monitoring Service of the European Union). First, using default settings we have drawn buffer zones with 1 and 10 km radius around all sampling points in Quantum GIS (QGIS) version 3.4 (QGIS Development Team, 2019). Following this, we constructed zonal histograms using the Processing Toolbox of QGIS. Finally, we calculated cover percentages for the buffer zones in R (version 3.4.1, R Core Team, 2017). From the 44 landscape classes present in the original CLC database, 28 were present in the buffer zones of our sampling points. We estimated land cover for each of these categories, but we subsequently performed a categorization of these in order to reduce dimensionality in the analyses. We considered watercourses and water bodies as unsuitable places for orchids. We did not join the different forest types, since broad‐leaved forest serves as habitats for some species which would not prefer shady coniferous forests; thus, different species have different forest type needs. We further considered vineyards, fruit trees, berry plantations, and land principally occupied by agriculture with significant areas of natural vegetation as “semi‐agricultural areas,” because when they are abandoned or extensively used, they could serve as orchid habitats. Details of the categorization are given in Table S1.

### 2.3 Statistical analyses

Statistical analyses were carried out in the R statistical environment (version 3.4.1, R Core Team, 2017).

To avoid multicollinearity in the models, we performed VIF selection (Craney & Surles, 2002) using vif function in fmsb package (Nakazawa, 2017) which calculates the VIF values for all of our explanatory variables, then removes the variable with the highest value, and repeats this until all VIF values are below the threshold, which in our case was “2.” Following this, the concerned variables were removed from the analyses. After testing the distribution of the data, we built generalized linear mixed models (GLMM) with quasi‐Poisson distribution due to significant overdispersion in the independent variables, using the *glmmPQL* function (*MASS* R package, Ripley et al., 2013). In all cases, we started by building full models containing all explanatory variables which were selected by VIF. This was followed by model simplification, when predictors were removed from the model using a stepwise backward procedure based on the largest p values. All predictors with *p* < 0.1 were retained in the minimal model. In all of the models, we used “country” and “sampling type (thematic or non‐thematic)” as random factors. Altogether, eight models were built with the following dependent variables: total number of species, total number of individuals, number of grassland species, number of forest species, number of species with broad ecological tolerance, number of individuals of forest, grassland, and broad ecological tolerance species. RMSE (root‐mean‐square error) values were calculated using *RMSE* function in the *performance* package (Lüdecke et al., 2019), and pseudo R^2^ values were calculated using the *r.squaredGLMM* function in the *MuMIn* package (Barton & Barton, 2019). Species were categorized into grassland, forest, and broad ecological tolerance categories using habitat descriptions of Delforge (2006) (Table S2.). Explanatory variables were Urban areas; Agricultural areas; Semi‐agricultural areas; Natural grasslands and pastures; Shrublands; Beaches, dunes, and sand plains; Sparsely vegetated areas; Wetlands; Broad‐leaved forests; Mixed forests (composed principally of trees, including shrub and bush understorey, where neither broad‐leaved nor coniferous species predominate); and Natural unsuitable places for vegetation within the 1‐km‐radius circle (the variable Coniferous forests was highly correlated with several land cover types and was thus eliminated from multivariate analyses by VIF). Another eight models were built with the same variables using 10‐km‐radius circle data (Table S3).

## 3 RESULTS

### 3.1 General results

**FIGURE 1:**
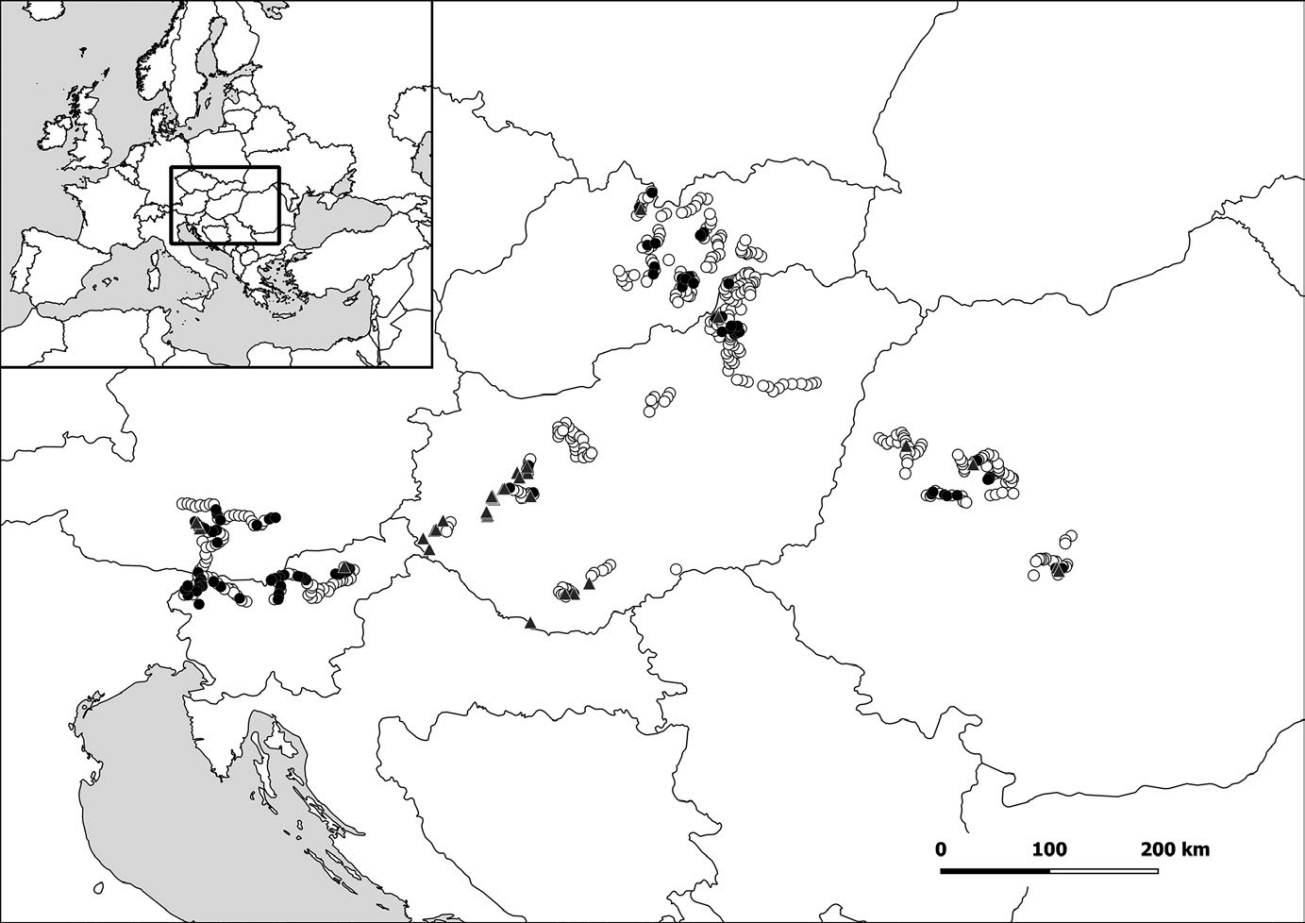
Distribution of thematic and non‐thematic sampling points in the surveyed countries. Gray triangles indicate non‐thematic sampling points; black dots indicate thematic sampling points with orchid presence, while white dots indicate the thematic sampling points with orchid absence

Out of the 465 thematic sampling points, we found orchids at 83 locations, with records in all of the five surveyed countries (Figure 1.). The ratio of the sampling points where orchids were present was the highest in Slovenia and the lowest in Hungary (Table 2.). Altogether, we found 2,272 orchid individuals belonging to 27 species (among these, 324 individuals could not be identified at the species level belonging to the genera *Epipactis* and *Platanthera*).

**TABLE 2:** Summary of survey data regarding proportion of thematic sampling points with orchid presence, as well as the overall number of species and number of individuals found at these locations across the five surveyed countries

Country	Ratio of thematic sampling points with orchid presence	Number of species	Number of individuals
Austria	30%	10	940
Hungary	9%	14	343
Romania	12%	10	351
Slovakia	15%	10	288

The most abundant species with 801 individuals was *Dactylorhiza fuchsii*, which was present in four of the five countries. It was followed by *Gymnadenia conopsea* with 320 individuals found in three countries. *Neottia ovata* was the only species found in all five countries, at nine localities with 67 individuals (Table S2). The rarest were two locally distributed *Gymnadenia* taxa, *Gymnadenia* × *suavolens* and *Gymnadenia lithopolitanica*. Among the species, there were 14 grassland specialists, seven forest specialists, and seven species characterized with broad ecological tolerance (Table S2).

Using the non‐thematic sampling protocol, at two sampling points in Austria, we found 23 individuals belonging to four species, including the rare and local *G. lithopolitanica*. In Hungary, 272 individuals were found belonging to 13 species at 33 non‐thematic sampling points. In Romania, 53 individuals from four species were found at four sampling points. In Slovakia and Slovenia, one non‐thematic locality was surveyed in both countries. In Slovakia, nine individuals were found from the species *Orchis mascula*, and in Slovenia, 61 individuals were found from two species (*D. fuchsii* and *Epipactis helleborine*) during non‐thematic surveys.

### 3.2 Landscape analyses

**FIGURE 2:**
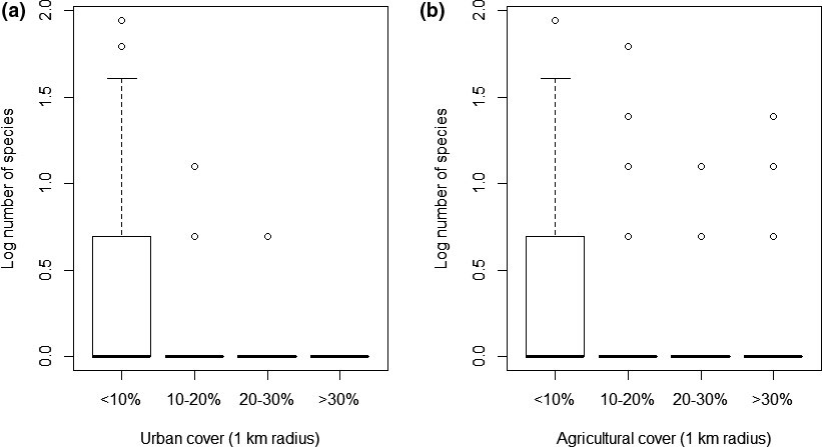
(a) Box plots showing the cover of urban areas within 1‐km‐radius circle and the logarithmized number of species in different coverage categories. (b) Box plots showing the cover of agricultural areas within 1‐km‐radius circle and the logarithmized number of species in different coverage categories

Multivariate models indicated a significant lower number of orchid species (Figure 2a,b) and individuals in sampling locations where the area of urban and agricultural land covers was higher within a 1 km radius (Table 3.). In the case of the total number of species, the cover of natural grasslands and pastures and broad‐leaved forest also had a significant negative effect.

**TABLE 3:** The eight minimal models (GLMMPQL) explaining variance in the number of species (left) and number of individuals (right) in overall, in grassland specialist and forest specialist and broad ecological tolerance orchids, respectively, in function of land cover within a 1 km radius

	Total number of orchid species				Total number of orchid individuals			
	*β*	*SE*	*t*	*p*	*β*	*SE*	*t*	*p*
Intercept	−1.00	0.33	−3.07	0.002	2.60	1.85	1.41	0.160
Urban areas	−0.80	0.23	−3.45	0.001	−1.58	0.51	−3.09	0.002
Agricultural areas	−0.66	0.15	−4.44	0.001	−0.65	0.26	−2.49	0.013
Natural grasslands and pastures	−0.27	0.10	−2.67	0.008				
Broad‐leaved forests	−0.24	0.12	−1.98	0.048				
Shrubland					0.17	0.09	1.99	0.047
Semi‐agricultural areas	−0.19	0.11	−1.69	0.091				
DF = 488 RMSE = 2.374, *R* ^2^c = 0.602					DF = 490 RMSE = 3.825, *R* ^2^c = 0.999			

	Number of grassland‐specialist orchid species				Number of grassland‐specialist orchid individuals			
	*β*	*SE*	*t*	*p*	*β*	*SE*	*t*	*p*
Intercept	−2.55	0.53	−4.81	0.001	−1.27	0.69	−1.85	0.065
Urban areas					−1.56	0.83	−1.89	0.060
Natural grasslands and pastures	0.40	0.15	2.60	0.010	0.73	0.15	4.89	0.001
Shrublands	0.18	0.11	1.66	0.097	0.51	0.12	4.16	0.001
Sparsely vegetated areas	0.14	0.07	1.94	0.053				
Mixed forests	0.42	0.17	2.46	0.014				
DF = 489 RMSE = 7.321, *R* ^2^c = 0.999					DF = 490 RMSE = 15.890 *R* ^2^c = 0.999			

	Number of forest‐specialist orchid species				Number of forest‐specialist orchid individuals			
	*β*	*SE*	*t*	*p*	*β*	*SE*	*t*	*p*
Intercept	−1.05	2.69	−0.39	0.696	−1.01	0.45	−2.25	0.025
Urban areas	−0.80	0.47	−1.71	0.088				
Broad‐leaved forests	0.70	0.20	3.48	0.001	0.84	0.20	4.20	0.001
DF = 491 RMSE = 6.264, *R* ^2^c = 0.418					DF = 492, RMSE = 3.437, *R* ^2^c = 0.987			

	Number of orchid species with broad ecological tolerance				Number of orchid individuals with broad ecological tolerance			
	*β*	*SE*	*t*	*p*	*β*	*SE*	*t*	*p*
Intercept	−2.03	0.38	−5.31	0.001	12.00	12.39	0.97	0.333
Urban areas	−0.94	0.30	−3.15	0.002	−2.18	0.90	−2.44	0.015
Agricultural areas	−0.83	0.20	−4.20	0.001	−0.70	0.30	−2.31	0.021
Natural grasslands and pastures	−0.55	0.14	−3.99	0.001	−0.76	0.18	−4.34	0.001
Broad‐leaved forests	−0.58	0.16	−3.73	0.001				
Mixed forests	−0.24	0.11	−2.17	0.031	−0.68	0.25	−2.77	0.006
DF = 488 RMSE = 3.027, *R* ^2^c = 0.081					DF = 489 RMSE = 19.601, *R* ^2^c = 0.999			

**FIGURE 3:**
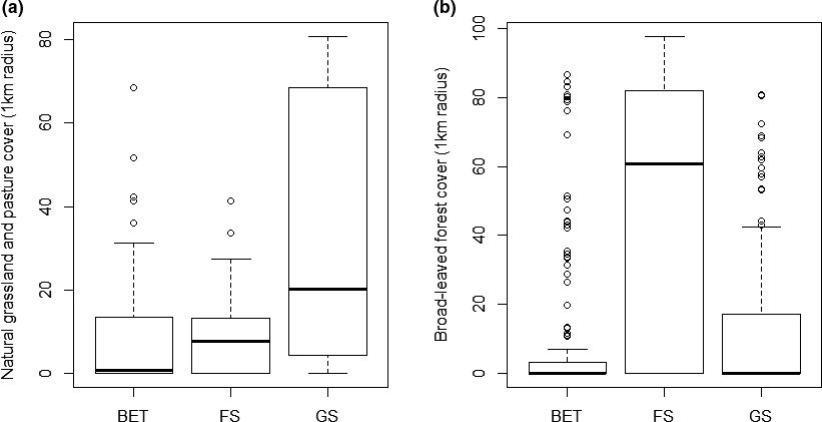
(a) Coverage of natural grassland and pasture within 1 km radius in the three orchid groups: species with broad ecological tolerance (BET), forest species (FS), and grassland species (GS). (b) Coverage of broad‐leaved forest within 1 km radius in the three groups: species with broad ecological tolerance (BET), forest species (FS), and grassland species (GS)

In the 1‐km‐radius circle, the cover of natural grasslands and pastures had a significant positive effect on the number of grassland species (Figure 3a) and number of grassland individuals. Shrublands also had a positive effect on both variables, but it showed marginal significance in the case of the number of individuals. Both in the case of number of forest orchid species (Figure 3b) and number of individuals belonging to these, the multivariate models indicated a significant positive effect on the land cover occupied by broad‐leaved forest.

We found a significant negative effect of the cover of urban and agricultural areas, natural grasslands and pastures, and mixed forest both on the number of species and individuals with broad ecological tolerance.

## 4 DISCUSSION

During our extensive field surveys, we found more than 2,000 orchid individuals of 27 different orchid species in roadsides. This alone suggests that roadsides provide an important habitat for orchids in Central Europe, similarly to other regions across Europe (Fekete et al., 2017, 2019). Furthermore, we found a number of rare orchid taxa on roadsides, including *G*. × *suaveolens* in Austria, *Orchis mascula* in Slovakia, a species that is near threatened according to the Red List of vascular plants of the Carpathian part of Slovakia (Turis et al., 2014). Additionally, we documented *Platanthera chlorantha* in roadsides of Hungary, a species listed as near threatened according to the Red List of the vascular flora of Hungary (Király, 2007). Overall, our surveys indicate that roadsides serve as suitable habitats for endangered taxa (according to IUCN Red List), such as *G. lithopolitanica* (Rankou, 2011). The highest number of orchid individuals present on roadsides belonged to *D. fuchsii*, a species that is characterized by a broad ecological tolerance.

Roadsides surveyed here hosted almost twice as many grassland specialist orchid species as forest specialists (i.e., 13 and 7, respectively), which might potentially be explained by road maintenance practices, namely the regular mowing of roadsides. Due to the latter, vegetation on roadsides is usually less closed, while mowing is known to have positive effects on grassland orchids in other habitat types (Curtis, 1946; Janečková et al., 2006; Sletvold et al., 2010; Smith & Cross, 2016). Forest specialist orchid species were also present on roadsides surveyed, being represented by more than 200 individuals. This suggests that despite being less adapted for regular mowing, they are able to cope and maintain populations in these anthropogenically influenced habitats. Roadsides are narrow grassland fragments and could act as ecotones (representing mainly transitions from grasslands to forest edges). Due to their weak competitive ability, orchids are frequently found in transitional, ecotone habitats, such as mesoxeric scrubland patches and forest edges (Bray & Wilson, 1992; Djordjević et al., 2016; Duchoň, 2012; Rai et al., 2010; Slaviero et al., 2016). Furthermore, they often colonize newly created habitat patches (such as roadsides), where the abundance of dominant plant species and the cover of trees and shrubs are low (Arditti & Ghani, 2000). Based on Grime's theory, orchids are considered as stress tolerators or ruderal species, (Dressler, 1981; Hágsater & Dumont, 1996) competing for resources and space, thus orchids around roadsides might compete for favorable light conditions, that are available at roadside verges, due to regular mowing (Djordjević & Tsiftsis, 2020).

Our multivariate models indicated a significant negative impact of agricultural and urban land covers on orchid diversity and abundance in general. Greater proportion of land cover occupied by urban and agricultural areas in the landscape matrix resulted in a lower number of orchid species and individuals present on roadsides. These land cover variables also had significant negative effects on the number of species with broad ecological tolerance and the number of individuals belonging to these species. Earlier study has already indicated that the surrounding landscape matrix has a high impact on species composition of habitats along roads (Tikka et al., 2000). Moreover, a decline in species richness on linear landscape fragments with increasing distance from seminatural habitats was also reported (Cousins & Lindborg, 2008). A study discussing drainage ditches showed that the proximity of natural grasslands increased the value of grassland vegetation of the ditches subsequently filled for restoration purposes, suggesting that their vegetation is highly dependent on the landscape matrix (Valkó et al., 2017).

We found that grassland specialists are mostly present on verges, where there are suitable grassland habitats adjacent to the roadside, while forest specialist is more common on roadsides where there are forests in the surrounding landscape. It is important to note that these linear landscape elements are often highly influenced by agricultural activities (e.g., use of fertilizers and herbicides) on adjacent fields (van Dorp, 1996); thus, species of nutrient‐poor ecosystems (such as orchids) are particularly unlikely to migrate along these elements (van Dorp et al., 1997; Thiele et al., 2018). When the landscape matrix environment is unsuitable for the dispersal of plants along ecological corridors, or dispersal is ineffective due to a high percentage of low‐quality patches, it is unclear whether they could truly function as a corridor. Under such circumstances, roadside patches might rather serve as refugia (van Dorp, 1996; van Dorp et al., 1997). Although according to Forman (1991) plants may in theory migrate along ecological corridors, there has been little empirical support for this; thus, it is more likely that the dispersal of native and rare plants occurring along the linear landscape elements is saltatory. However, the spread of less sensitive, successful alien and invasive species along roads is a well‐known phenomenon (Benedetti & Morelli, 2017; Dar et al., 2018; Vakhlamova et al., 2016). This is an especially likely scenario in the case of orchids since their microscopic seeds are effectively dispersed by the wind, even over long distances (Arditti & Ghani, 2000), thus facilitating the effective colonization of new habitat patches. This is in correspondence with another study, suggesting that corridor use is common mostly in case of plant species that lack the ability of long‐distance dispersal (Thiele et al., 2018). Nonetheless, the conservation importance of roadsides is not to be underrated due to these facts, since these corridors most likely still function as linear reserves for plants (Forman, 1991). Consequently, the conservation value of these narrow fragments of seminatural habitats is becoming increasingly appreciated worldwide (Bernes et al., 2017; Hopper, 1990; Melman & Verkaar, 1991; Niu et al., 2019; Ryttäri & Kettunen, 1997; Saunders & Hobbs, 1991), and in some countries, they have been identified as Sites of Special Scientific Interest (Parr & Way, 1988).

Overall, it is becoming clear that plants are able to disperse to roadsides from the surrounding landscape, but the possibility exists that colonizations might occur the other way around as well, which could facilitate the natural restoration of degraded grasslands adjacent to roads. Moreover, roadsides with properly managed native vegetation could contribute to pollinator conservation, which is particularly important today, as we are facing a global pollination crisis (Hopwood, 2008; Hopwood et al., 2015; Wojcik & Buchmann, 2012). Many orchids are specialist species; therefore, their conservation is important because their disappearance leads to functional homogenization in ecosystems, promoting biodiversity loss (Clavel et al., 2011). Thus, orchids might serve as general indicators of the ecological state of roadside vegetation. Moreover, considering tendencies of decline in seminatural habitats worldwide, it is possible that roadsides will serve as important refugia that could aid the maintenance of floristic diversity.

## 5 IMPLICATIONS FOR MANAGEMENT

Here, we emphasize that floristic surveys of roadsides and adjacent areas are key for planning appropriate road management. We believe that management planning should be conducted in accordance with local and regional conservation efforts, because roadside vegetation and its importance changes along the roads, depending on the habitats they pass through. Appropriate planning, building, and management of roads should focus on creating and maintaining roadsides in states that are suitable for natural vegetation. Generally speaking, during planning, it is desirable to avoid creating steep or concrete retaining walls; gentle slopes should be established instead, in order to form a gradual transition to the natural landform. Terracing with rock outcrops can support this by facilitating the establishment of vegetation and by creating microclimatic niches, while they stabilize the structure of road cuttings (Iuell et al., 2003). Whenever possible and when more time is available for the stabilization of verges, during road building or broadening, the use of subsoil—instead of topsoil—would be favorable for the reduction of soil fertility, since high fertility negatively affects the floristic composition of natural grasslands (Gough & Marrs, 1990). Local origin of the soil used during construction is also very important, as soils from a different source can contain seeds of alien species (Greenberg et al., 1997). To facilitate floristic and pollinator diversity after construction, it is also favorable to revegetate roadsides using specific seed mixtures appropriate for adjacent vegetation. Pollinators are key factors in the maintenance of native vegetation on roadsides; thus, it is very important to reduce their collision with cars, by keeping the meadows a few meters away from the road's edge and keeping long continuous flower meadows, reducing their will to cross the road for flowering patches (Hopwood et al., 2010; Keilsohn et al., 2018). As a part of roadside management, regular mowing is a cost‐efficient element of their maintenance, and it is obligatory in most of the countries for safety reasons. According to a previous study from the Mediterranean, the regularly mowed 0–2‐m part of the roadside is the most suitable for orchid individuals (Fekete et al., 2019). However, there could be a difference in regularity of roadside mowing due to climatic differences between the Mediterranean and the European roadside verges. In the Mediterranean region, the growth of the vegetation could be slower, while the best practice for creating and maintaining species‐rich meadows along European roads should be mowing twice per year (this being better compared to once a year), and the hay should be removed after each cutting (Jakobsson et al., 2018). The use of herbicides and paving of roadsides is strongly unadvised. We further urge local authorities to conduct appropriate field surveys and impact assessments before broadening roads.

## CONFLICT OF INTEREST

The authors declare that they have no known competing financial interests or personal relationships that could have appeared to influence the work reported in this paper.

## AUTHOR CONTRIBUTIONS

**Réka Fekete:** Conceptualization (equal); data curation (equal); formal analysis (equal); writing – original draft (lead). **Judit Bódis:** Investigation (equal); writing – review and editing (supporting). **Bence Fülöp:** Investigation (equal). **Kristóf Süveges:** Investigation (equal). **Renáta Urgyán:** Investigation (equal). **Tamás Malkócs:** Formal analysis (equal); writing – review & editing (supporting). **Orsolya Vincze:** Formal analysis (lead); methodology (equal); writing – review & editing (equal). **Luís Silva:** Conceptualization (equal); formal analysis (lead). **Attila Molnár V.:** Conceptualization (equal); supervision (equal).

## Supporting information

Appendix S1

## Data Availability

All sample data used in the analyses are available from Dryad at https://doi.org/10.5061/dryad.tdz08kpxv.
