# Supplementary material for: Roadsides provide refuge for orchids: characteristic of the surrounding landscape
Source: Ecol Evol. 2020 Oct 26;10(23):13236–47. doi: 10.1002/ece3.6920 (PMC7713965; doi:10.1002/ece3.6920)
Supplement: Supplementary file 1 — Appendix S1 [file ECE3-10-13236-s001.docx]

**Appendix S1**

**Table S1. Original landscape classes according to Copernicus and our categories used in the analyses.**

| **Categories used in the analyses** | **Original land cover categories** |
| --- | --- |
| Urban areas | Continuous urban fabric, Discontinuous urban fabric, Industrial or commercial units, Road and rail networks and associated land, Airports, Mineral extraction sites, Dump sites, Construction sites, Sport and leisure facilities |
| Agricultural areas | Non-irrigated arable land, Complex cultivation patterns |
| Semi-agricultural areas | Vineyards, Fruit trees and berry plantations, Land principally occupied by agriculture with significant areas of natural vegetation |
| Natural grasslands and pastures | Natural grasslands, Pastures |
| Shrublands | Moors and heathland, Transitional woodland shrub |
| Sparsely vegetated areas | Bare rocks, Sparsely vegetated areas |
| Wetlands | Inland marshes, Peatbogs |
| Unsuitable places for orchids | Water courses, Water bodies |
| Beaches, dunes, and sand plains | Beaches, dunes, and sand plains |
| Broad-leaved forests | Broad-leaved forest |
| Mixed forests | Mixed forest |
| Coniferous forests | Coniferous forest |

**Table S2. Details of the species found at the roadside verges in the surveyed countries.**

| **Species** | **Category based on light requirements (Delforge 2006)** | **Number of individuals** | **Number of sampling points** | **Countries where present** |
| --- | --- | --- | --- | --- |
| *Anacamptis pyramidalis* (L.) L.C.M. Richard | grassland | 53 | 2 | Hungary, Slovakia |
| *Cephalanthera damasonium*  (Miller) Druce | forest | 42 | 7 | Hungary, Romania, Slovakia |
| *Cephalanthera longifolia* (L.) Fritsch | forest | 116 | 13 | Hungary, Romania, Slovakia, Slovenia |
| *Cephalanthera rubra* (L.) L.C.M. Richard | forest | 16 | 5 | Romania, Slovenia |
| *Coeloglossum viride* (L.)Hartman | grassland | 28 | 2 | Austria |
| *Corallorhiza trifida* Châtelain | forest | 4 | 2 | Slovenia |
| *Dactylorhiza fuchsii* (Druce) Soó nom. cons. prop | broad ecological tolerance | 801 | 79 | Austria, Romania, Slovakia, Slovenia |
|  |  |  |  |  |
| *Epipactis atrorubens* (Hoffmann ex Bernhardi) Besser | broad ecological tolerance | 115 | 10 | Austria, Romania, Slovenia |
| *Epipactis helleborine* (L.) Crantz | forest | 60 | 10 | Slovenia |
| *Epipactis microphylla* (Ehrhardt) Schwartz | forest | 4 | 2 | Hungary, Romania |
| *Epipactis* sp. |  | 317 | 29 | Austria, Hungary, Romania, Slovakia, Slovenia |
| *Gymnadenia albida* (L.) L.C.M. Richard | grassland | 5 | 1 | Austria |
| *Gymnadenia austriaca* (Teppner and E. Klein) P. Delforge | grassland | 13 | 3 | Austria |
| *Gymnadenia conopsea* (L.) R. Brown | grassland | 320 | 9 | Austria, Romania, Slovenia |
| *Gymnadenia lithopolitanica* (V. Ravnik)Teppner and E. Klein | grassland | 2 | 1 | Austria |
| *Gymnadenia odoratissima* (L.) L.C.M. Richard | grassland | 7 | 1 | Austria |
| *Gymnadenia x suaveolens* (Villars) Wettstein | grassland | 1 | 1 | Austria |
| *Himantoglossum adriaticum* H. Baumann | grassland | 85 | 7 | Hungary |
| *Neottia nidus-avis* (L.) L.C.M. Richard | forest | 6 | 3 | Hungary, Slovenia |
| *Neottia ovata* (L.)Bluff and Fingerhuth | broad ecological tolerance | 67 | 13 | Austria, Hungary, Romania, Slovakia, Slovenia |
| *Ophrys sphegodes* Miller | grassland | 13 | 1 | Hungary |
| *Orchis mascula* (L.) L. | broad ecological tolerance | 10 | 2 | Slovakia |
| *Orchis militaris* (L.) | grassland | 7 | 2 | Hungary, Slovakia |
| *Orchis morio* L. | grassland | 59 | 9 | Hungary |
| *Orchis purpurea* Hudson | broad ecological tolerance | 40 | 16 | Hungary |
| *Orchis simia* Lamarck | grassland | 29 | 4 | Hungary |
| *Platanthera bifolia* (L.) L.C.M. Richard | broad ecological tolerance | 32 | 5 | Hungary, Romania |
| *Platanthera chlorantha* (Custer) Reichenbach | broad ecological tolerance | 4 | 1 | Hungary |
| *Platanthera* sp. |  | 7 | 4 | Hungary, Slovakia, Slovenia |

**Table S3. The eight minimal models (GLMMPQL) explaining variance in the number of species (left) and number of individuals (right) in overall, in grassland-specialist and forest-specialist and broad-ecological tolerance orchids respectively in function of land cover within a 10 km radius.**

| **Total number of species** | | | | | **Total number of individuals** | | | |
| --- | --- | --- | --- | --- | --- | --- | --- | --- |
|  | β | SE | t | p | β | SE | t | p |
| **Intercept** | -0.83 | 0.35 | -2.40 | 0.017 | 2.30 | 1.30 | 1.77 | 0.076 |
| **Urban areas** | -0.30 | 0.13 | -2.38 | 0.018 | -0.76 | 0.28 | -2.67 | 0.008 |
| **Agricultural areas** | -0.53 | 0.17 | -3.21 | 0.001 |  |  |  |  |
| **Natural grasslands and pastures** | -0.21 | 0.12 | -1.79 | 0.075 | 0.34 | 0.15 | 2.30 | 0.022 |
| **Shrubland** | 0.21 | 0.07 | 2.95 | 0.003 | 0.44 | 0.13 | 3.47 | p<0.001 |
| **Mixed forests** |  |  |  |  | 0.42 | 0.25 | 1.71 | 0.088 |
| **Number of grassland species** | | | | | **Number of grassland individuals** | | | |
|  | β | SE | t | p | β | SE | t | p |
| **Intercept** | -2.77 | 0.49 | -5.65 | p<0.001 | -1.23 | 0.73 | -1.68 | 0.093 |
| **Natural grasslands and pastures** | 0.50 | 0.16 | 3.11 | 0.002 | 1.07 | 0.21 | 5.09 | p<0.001 |
| **Shrubland** | 0.61 | 0.12 | 4.99 | p<0.001 | 0.80 | 0.28 | 2.86 | 0.004 |
| **Number of forest species** | | | | | **Number of forest individuals** | | | |
|  | β | SE | t | p | β | SE | t | p |
| Intercept | -2.88 | 0.50 | -5.72 | p<0.001 | -1.42 | 0.63 | -2.25 | 0.025 |
| Agricultural areas | -0.42 | 0.21 | -2.06 | 0.0403 |  |  |  |  |
| Natural grasslands and pastures | -1.14 | 0.28 | -4.07 | p<0.001 | -1.66 | 0.38 | -4.35 | p<0.001 |
| Broad-leaved forests | 0.51 | 0.20 | 2.59 | 0.0099 | 0.78 | 0.23 | 3.36 | 0.001 |
| **Number of species with broad ecological tolerance** | | | | | **Number of individuals with broad ecological tolerance** | | | |
|  | β | SE | t | p | β | SE | t | p |
| Intercept | -1.97 | 0.40 | -4.92 | p<0.001 | 0.28 | 0.56 | 0.50 | 0.620 |
| Urban areas | -0.65 | 0.18 | -3.59 | 0.000 | -0.66 | 0.29 | -2.26 | 0.024 |
| Agricultural areas | -0.50 | 0.20 | -2.53 | 0.012 | -0.65 | 0.39 | -1.67 | 0.096 |
| Natural grasslands and pastures | -0.31 | 0.15 | -2.05 | 0.041 |  |  |  |  |
| Broad-leaved forests | -0.56 | 0.20 | -2.86 | 0.005 | -0.64 | 0.31 | -2.08 | 0.038 |
| Unsuitable places for vegetation | 0.23 | 0.09 | 2.69 | 0.008 | 0.25 | 0.11 | 2.35 | 0.019 |
